# Supplementary material for: Printable homocomposite hydrogels with synergistically reinforced molecular-colloidal networks
Source: Nat Commun. 2021 May 14;12:2834. doi: 10.1038/s41467-021-23098-9 (PMC8121785; doi:10.1038/s41467-021-23098-9)
Supplement: Supplementary file 1 — Supplementary Information [file 41467_2021_23098_MOESM1_ESM.pdf]

Supplementary Information

Printable homocomposite hydrogels with synergistically reinforced molecular-colloidal networks

Austin H. Williams, Sangchul Roh, Alan R. Jacob, Simeon D. Stoyanov,
Lilian Hsiao and Orlin D. Velev*

**E-mail: odvelev@ncsu.edu*

Supplementary Discussion

Alginate Hydrogel Soft Dendritic Colloid Fabrication

In order to make the nanofibrous reinforcement for HHGs presented herein, we synthesized sodium alginate (SA) soft dendritic colloids (SDCs) with hierarchical, branched micro- and nanofibers. SA SDCs are fabricated using shear-driven polymer precipitation within a nonsolvent flow of an aqueous $CaCl_2$ solution. Upon injection of the SA solution into the turbulently sheared precipitation medium, the solution is randomly and vigorously stretched and templated by the hierarchical turbulent eddies of the Ca^{2+} rich medium. Concurrent with the multidirectional deformation of the polymer solution by the turbulent flow, the Ca^{2+} ions rapidly diffuse into the stretched SA solution droplet. The simultaneous droplet elongation by turbulent eddies and Ca^{2+} diffusion result in the precipitation of the alginate into highly branched and fibrous SA SDCs. While the morphology remains the same, these SDCs are fundamentally different from those of our previous report. Herein, we rely upon the rapid, ionic crosslink reaction between alginate and Ca^{2+} as the precipitation mechanism to form water-swollen and cross-linked SA SDC fibers. Contrarily, our previous report utilized nonsolvent-induced precipitation, which resulted in

similarly structured, but more densely packed polymer fibers. While the precipitation mechanisms of the two materials may differ, the resulting particulates of both processes possess the characteristic SDC morphology. We hypothesize that parameters such as temperature, pH, and exposure to light that can induce rapid precipitation in other polymer systems can also be utilized as precipitation mechanisms under shear to obtain SDCs from a vast library of materials.

Characterization of Alginate SDCs

SA SDC suspensions were washed with deionized water to remove redundant Ca^{2+} ions using centrifugation following by decanting and resuspension of the SDC pellet in deionized water. After rinsing, a dilute suspension of SDCs (< 0.001 wt.%) in ethanol was prepared and spin-coated on a silicon wafer and a glass slide to observe individual SA SDCs with a field-emission scanning electron microscopy and optical microscopy (Fig. 1a). SDCs can often be particulates spanning hundreds of microns in total diameter (Figure 1b), however, the backbone fibers comprising the greater particulate branch hierarchically until the outer layer ("corona") consists of thin flexible nanofibers whose networking is dominated by physical interactions and fiber entanglements.

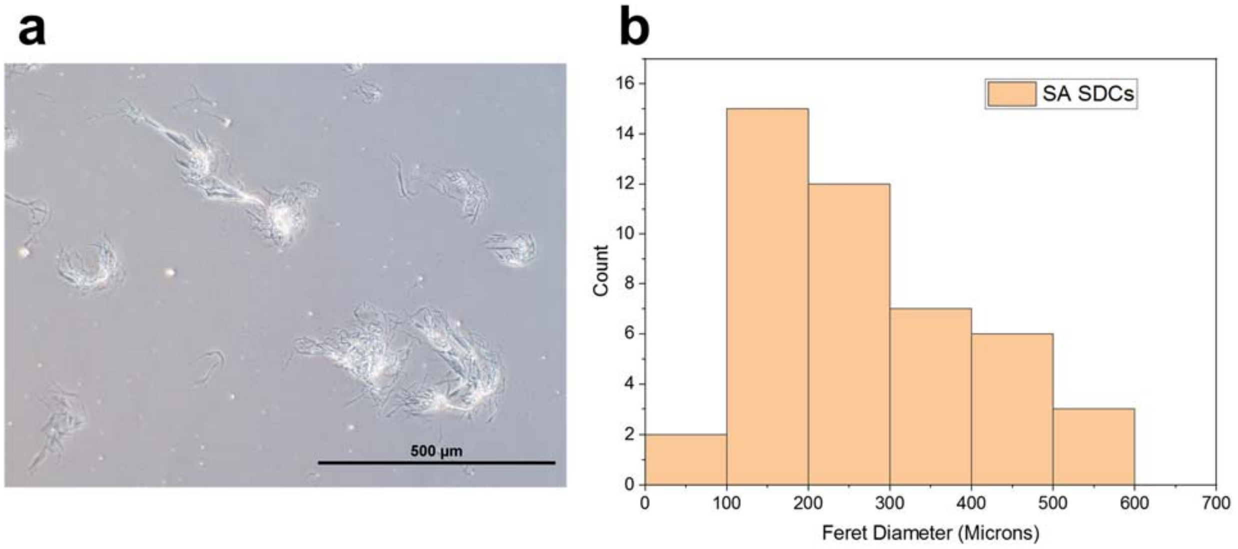

Supplementary Figure 1 | Size distribution of SA SDCs. **a** Phase contrast optical micrograph of SA SDCs spin-coated onto a glass slide. **b** Histogram showing the Feret Diameter size distribution of the coronas of nanofibers around the dried SDCs obtained from microscope images (n = 45).

The pictures of the SDC solutions in Supplementary Fig. 2 show that while suspensions of SA SDCs show gel-like behavior when analyzed using a frequency sweep down to concentrations of 0.25 wt.%, these dilute suspensions flow upon inversion of their vials until a concentration between 0.50 – 0.75 wt.%. The impressive network-building capabilities by SDC suspensions are evidenced by their yield stress behavior at dilute concentrations as low as 0.25 wt.% (Supplementary Fig. 2c), while non-cross-linked SA solutions show Newtonian behavior up to concentrations of 3 wt.%. Interestingly, the SDC suspension at just 0.25 wt.% has a complex modulus nearly matching that of the 3 wt.% SA solution containing 12× the alginate.

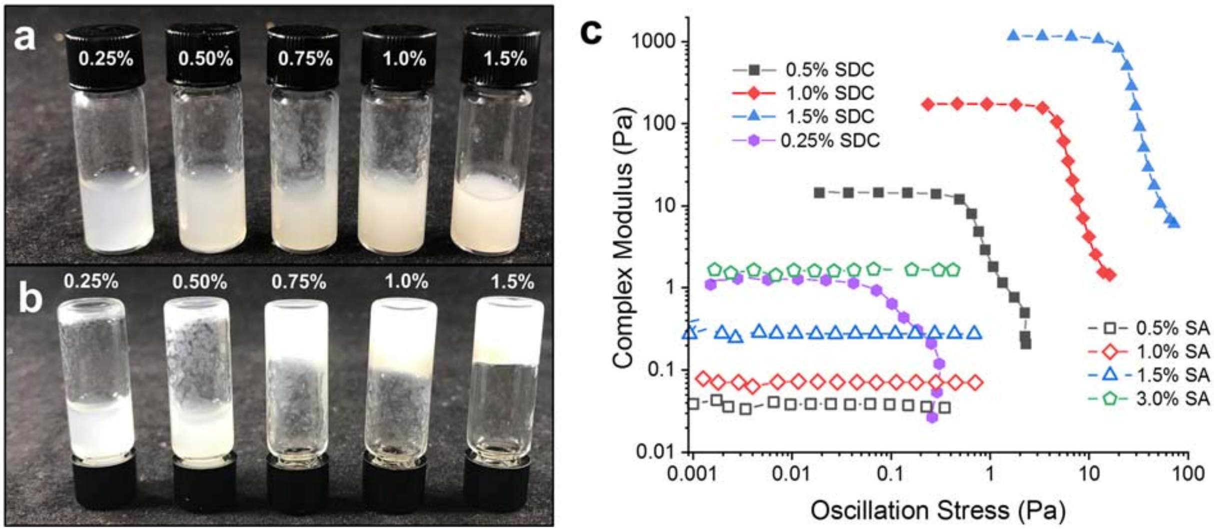

Supplementary Figure 2 | Gelation of SA SDC suspensions. Pictures of aqueous SA SDC suspensions at concentrations ranging 0.25 – 1.5 wt.% **a** before and **b** after inversion of the vials indicating apparent yield stress and gel-like behavior with increasing concentration. **c** Amplitude sweeps performed on dissolved SA solutions (unfilled) and aqueous SA SDC suspensions (filled) of varied concentrations showing yield stress behavior in dilute SDC suspensions.

To determine the $CaCO_3$ loading capacity of the SA cross-linked molecular hydrogels (CMHs), a series of 1.0 wt.% SA gels were prepared with increasing $CaCO_3$ content and the D-Gluconic acid δ-lactone (GDL) added to the gels was raised proportionally with the $CaCO_3$ content (Supplementary Fig. 3a). These gels showed significant syneresis with increased $CaCO_3$ and GDL content, which was quantified by using image analysis to measure the decrease in the area of the gels over time within their 50 mm × 44 mm × 1.5 mm acrylic molds (Supplementary Fig. 3b). The SA CMHs containing high $CaCO_3$ loading (0.5 wt.% and 1.0 wt.%) do not become completely transparent after 24 hours indicating incomplete $CaCO_3$ dissolution and lose significant volume by syneresis. Next, a set of gels containing 1.0 wt.% SA, 0.5 wt.% $CaCO_3$, and varying concentrations of added GDL were prepared. It can be seen visually that even up to 140.7 mM GDL, the mixture retains its opacity, indicating incomplete dissolution of the $CaCO_3$ nanoparticles (Supplementary Fig 3c).

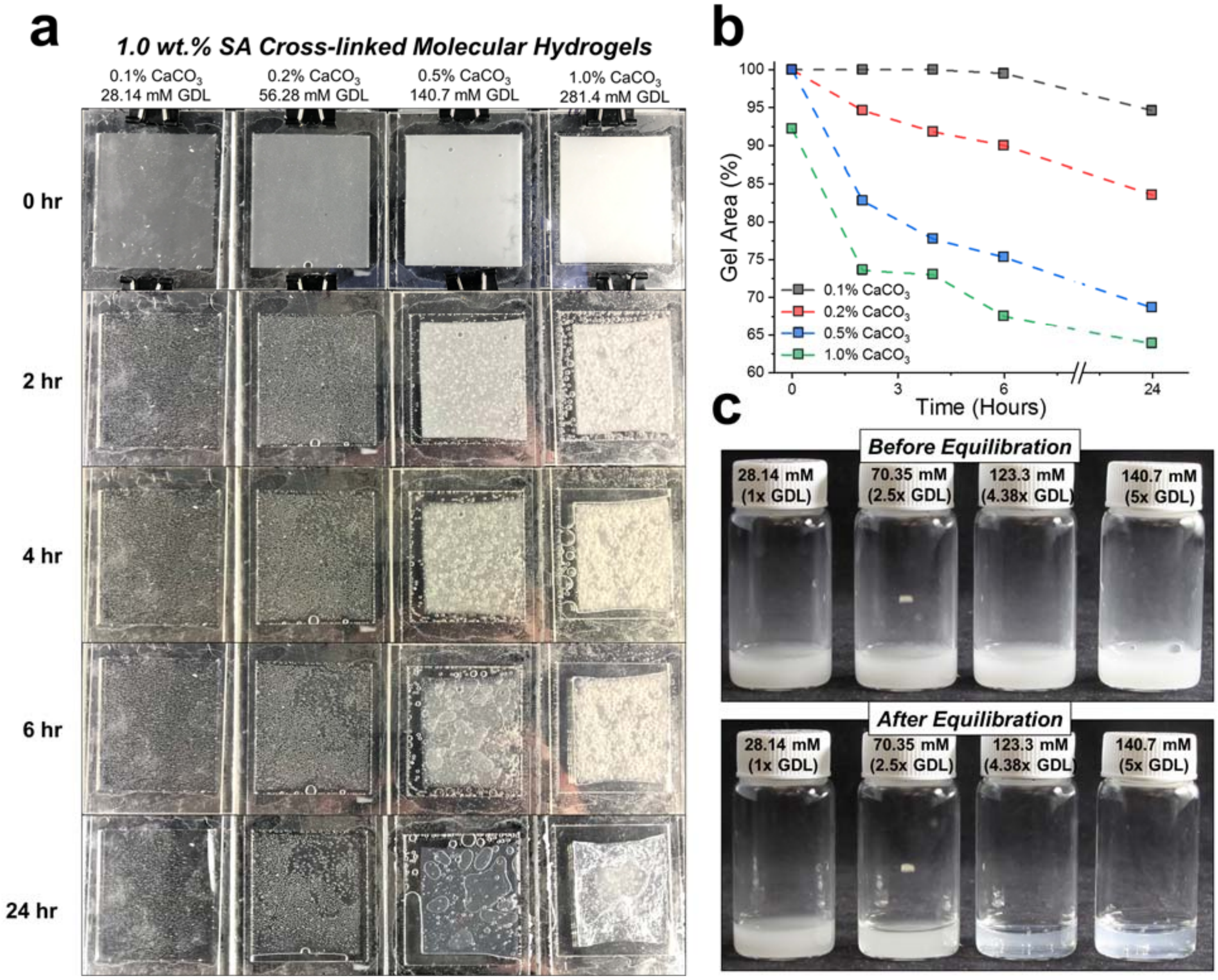

Supplementary Figure 3 | Syneresis of SA CMHs with increased $CaCO_3$ content. **a** Pictures of gel syneresis prepared in 50 mm × 44 mm × 1.5 mm sealed acrylic molds and **b** the area of the gels over time measured by image analysis in ImageJ of the area loss of the gels over time. **c** Pictures of 1.0 wt.% SA, 0.5 wt.% $CaCO_3$ mixtures immediately after addition of 28.14 – 140.7 mM GDL.

Elucidating the Role of Calcium in SDC Gelation

To determine the level of calcium incorporated into the fibers of the SA SDCs during fabrication, a 1.0 wt.% suspension of SA SDCs was repeatedly washed with deionized water to remove excess ions from solution and 8 mL of the suspension was deposited in an aluminum dish and dried at 50°C for 24 hours. A 1.0 wt.% molecular alginate gel was also prepared with 0.11 wt.% $CaCO_3$ NPs and 28.14 mM GDL. This mixture was then deposited in an aluminum weighing dish, allowed to gel, and placed in a 50°C oven for 24 hours to dry to produce a thin, cross-linked alginate film

of 10 wt.% $CaCO_3$. These films were placed under vacuum for 2 days and analyzed by X-ray photoelectron spectroscopy (XPS, Supplementary Fig. 4). The atomic percentage of calcium in the CMH film was determined to be 0.5 wt.%, while that of the SDC film was found to 2.6 wt.%, indicating that the SDCs contain approximately 5× the amount of calcium than the molecular alginate film at the prepared concentration.

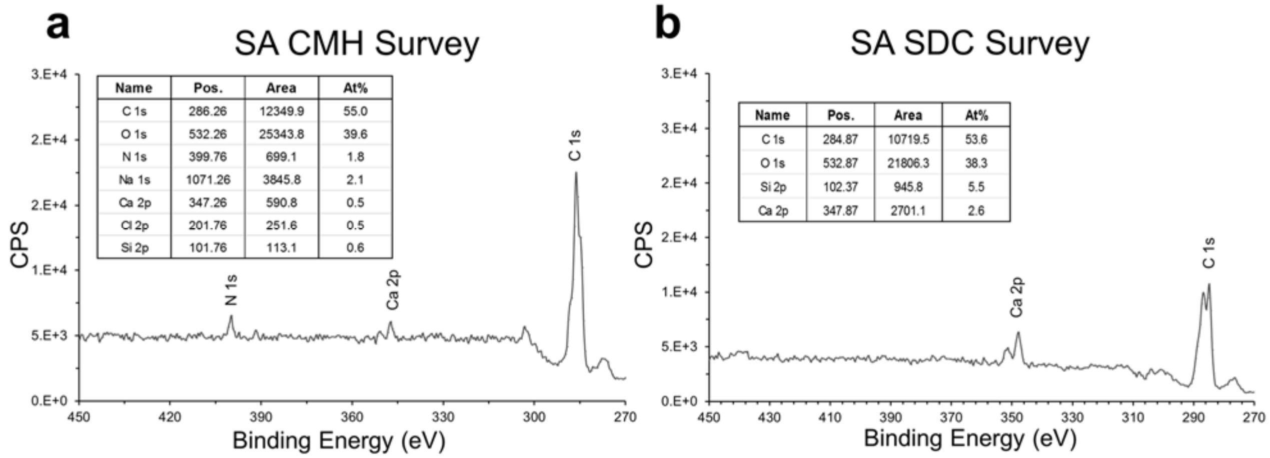

Supplementary Figure 4 | X-ray photoelectron spectroscopy analysis of dried molecular alginate and SDC films. a XPS spectrum of a dried molecular alginate film prepared with by drying a 1 wt.% SA, 0.10 wt.% $CaCO_3$ NPs, 28.14 mM GDL CMH into a film. **b** XPS spectrum of a dried SA SDC film after repeated washing an aqueous SDC suspension with deionized water and drying.

With SA SDCs containing appr. 5× the calcium content of the CMH, it is then possible to calculate the concentrations in [mM] of each constituent of the gel (Supplementary Tables 1 and 2). With the maximum HHG tensile stiffness resulting from HHGs with a composition of 0.125 wt.% SDC/0.875 wt.% (Fig. 3), it can then be estimated the calcium content of this HHG is appr. 15.2 mM, or 1.5× that of the pure CMH.

Supplementary Table 1. Equilibrium compositions of SA CMH/SDC HHGs in [wt.%].

SA CMH/SDC Homocomposite Compositions (wt.%)

**wt.% SDC**	**0.000**	**0.125**	**0.250**	**0.500**	**0.750**	**1.000**
**wt.% CMH**	**1.000**	**0.875**	**0.750**	**0.500**	**0.250**	**0.000**
$CaCO_3$ (wt.%)	0.100	0.0875	0.075	0.050	0.025	0.000
Total [Ca^{2+}] (wt.%)	0.040	0.061	0.082	0.124	0.166	0.208
[GDL] (wt.%)	0.500	0.439	0.378	0.250	0.125	0.000

Supplementary Table 2. Estimated equilibrium compositions of 1.0 wt.% SA CMH/SDC HHGs in [mM].

SA CMH/SDC Homocomposite Compositions (mM)

**wt.% SDC**	**0.000**	**0.125**	**0.250**	**0.500**	**0.750**	**1.000**
**wt.% CMH**	**1.000**	**0.875**	**0.750**	**0.500**	**0.250**	**0.000**
Total [Ca^{2+}] (mM)	9.991	15.236	20.482	30.972	41.463	51.953
[GDL] (mM)	28.143	24.625	21.107	14.071	7.036	0.000

Structural reversibility comparison of the hydrogel systems

To investigate the reversibility of the Calcium-alginate ionic cross-links in SA HHGs, 1.0 wt.% SA SDC, CMH, and HHG (0.5% SDC, 0.5% CMH) samples were treated with the chelating agent ethylenediaminetetraacetic acid (EDTA). A 1.0 wt.% SA SDC in water suspension was immersed in a 50 mM EDTA solution (Supplementary Fig. 5a). Immediately following the addition of the EDTA solution, a significant loss of turbidity in the suspension is observed as the EDTA sequesters the Ca^{2+} cross-links and the free alginate chains dissolve back into the aqueous phase, while the SDC suspension in pure water sees no appreciable visible change. Samples of 1.0 wt.% SA CMH

gel showed similar behavior in water and in EDTA solution (Supplementary Fig. 5b). While the CMH gel stored in water maintained its structure, the CMH sample treated with EDTA exhibited gel fragmentation after 10 minutes and, after 24 hours, nearly complete dissolution of the alginate back into the water phase. The SA SDCs may show more a more rapid degradation in response to EDTA addition because the greater surface area of SDCs in comparison to the CMH gel. Finally, the HHG gel treated with EDTA did show a decrease in gel volume after 24 hours, but maintained the original hydrogel structure, suggesting that the HHG samples may be less prone to gel breakdown by chelating agents (Supplementary Fig. 5c). Absent such strong chelating agents, we found that SDCs and HHGs stored in pure water are stable and maintain their distinct morphological features and rheological behavior after several months of storage at room temperature.

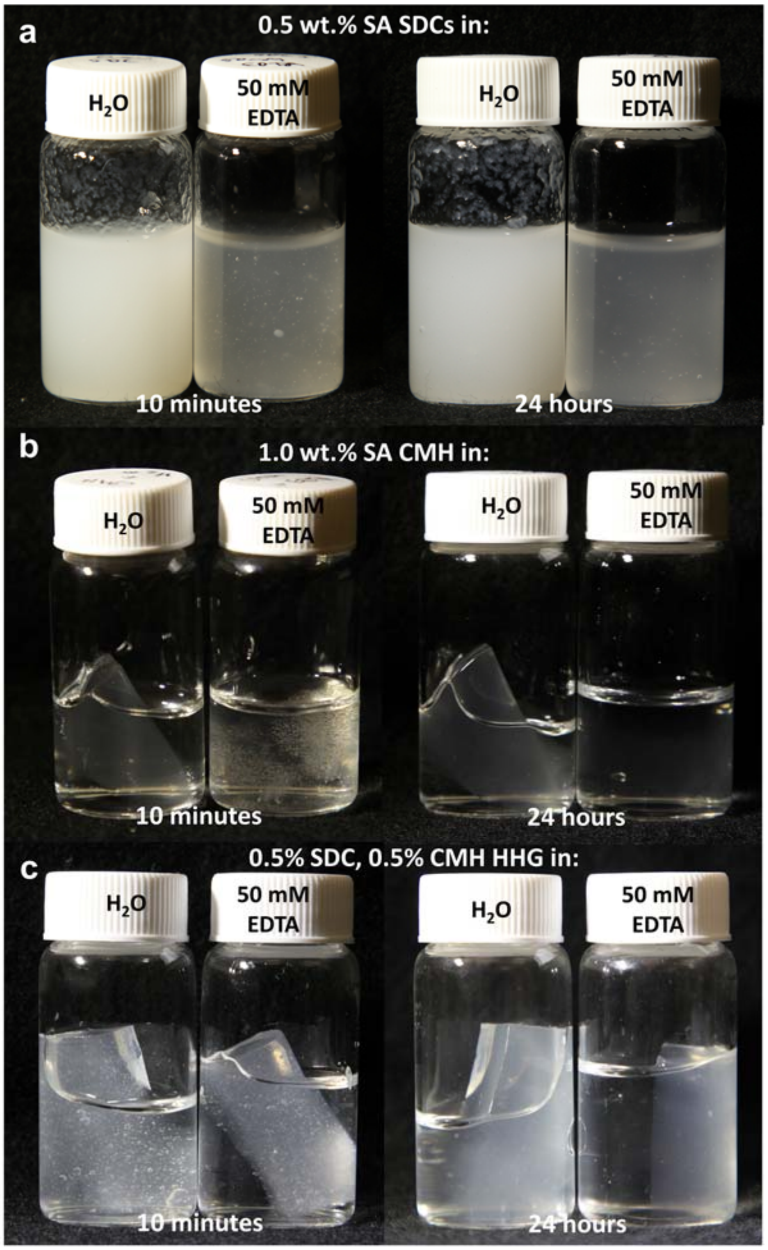

Supplementary Figure 5 | Degradation of SA SDC and CMH gels by chelating agents. a Pictures of 0.5 wt.% SA SDCs suspended in pure water and 50 mM EDTA after 10 minutes and 24 hours following 50mM EDTA solution addition. **b** Pictures of 1.0 wt.% SA CMH and **c** 1.0 wt.% HHG (0.5 wt.% SDC, 0.5 wt.% CMH) treated with pure water and 50 mM EDTA 10 minutes and 24 hours following addition of EDTA solution.

Rheological Time Sweep Analysis of SA CMHs and HHGs

To ensure that the ionization of $CaCO_3$ NPs by the GDL-mediated pH drop was solely responsible for the gelation seen in CMH and homocomposite hydrogel samples, a series of time sweep

experiments were performed in which only GDL as well as $CaCO_3$ and GDL were added to pure SA CMH and SDC samples. When the combination of $CaCO_3$ NPs and GDL were added to a dissolved alginate (SA in H_2O) solution, the sample saw gelation as expected. Without the presence of the $CaCO_3$ NPs, the SA solution did not see any gelation despite the drop in the solution pH from 8.1 to 3.6 after completing equilibration (Supplementary Fig. 6a).

The same experiments were performed on a 0.67 wt.% SA SDC suspension in deionized water and it was found that the addition of $CaCO_3$ did increase the storage and loss moduli of the suspension slightly initially, however, the dissolution of those $CaCO_3$ NPs over time by GDL hydrolysis led to the weakening of these suspensions and eventually, fluid-like behavior with the loss modulus eclipsing the storage modulus (Supplementary Fig. 6b). This result indicates that the SA SDCs are unable to be further cross-linked in suspension by the introduction of Ca^{2+} ions *in situ*. Furthermore, the addition of solely GDL to the SDC suspension resulted in the weakening of the suspension over time. The combination of these results with the CMH and SDC systems shows that the greatly increased moduli seen in the homocomposite hydrogels is not a byproduct of any GDL-mediated pH drop or tertiary cross-linking phenomena between the SDCs and the $CaCO_3$-GDL combination, but instead, is a result of the interplay between the molecular alginate network of the CMH and the colloidal alginate network of the SDCs.

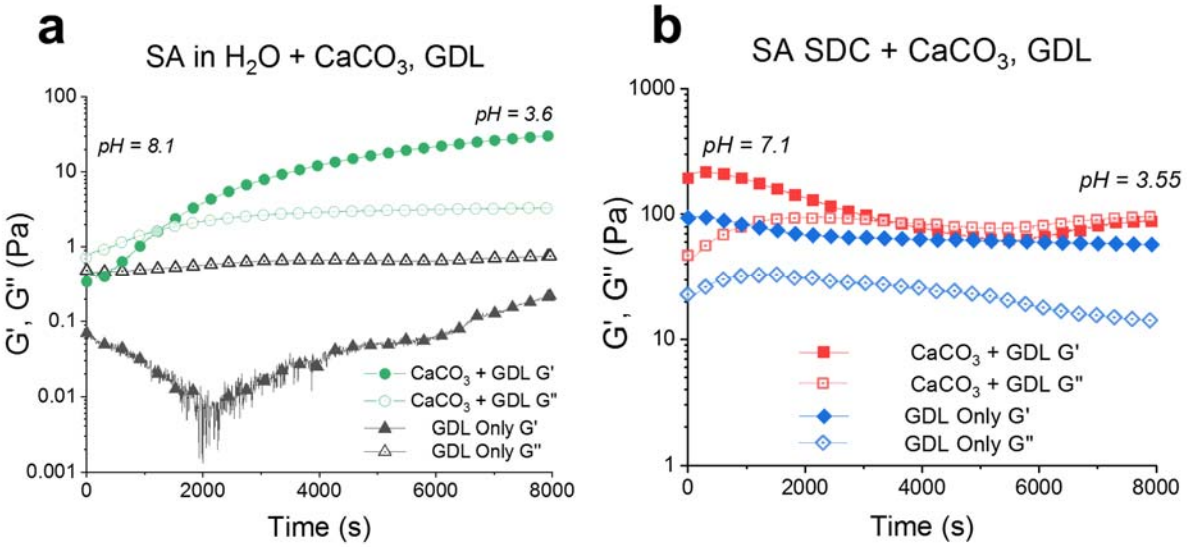

Supplementary Figure 6 | Time sweeps of SA SDCs and molecular alginate solutions (SA in H_2O) with addition of $CaCO_3$ and GDL. a Time sweeps of 1.0 wt.% SA SDC suspensions with and without 0.1 wt.% $CaCO_3$ NPs following addition of 28.14 mM GDL. **b** Time sweeps of 0.67 wt.% SA SDC suspensions with and without 0.067 wt.% $CaCO_3$ NPs following addition of 18.77 mM GDL. Both graphs show the pH of the suspensions and solutions before and after GDL addition and equilibration.

Rheological analysis of the 1.0 total wt.% HHGs following equilibration (Supplementary Fig. 7) shows the maximum complex modulus of the 1.0 total wt.% equilibrated alginate HHGs resulted at a 1/1 SDC/CMH ratio, despite the maximum stiffness being obtained at lower SDC concentrations. A maximum complex modulus being obtained at 1/1 a SDC/CMH formulation is seen again in Figure 5b with the 1.5 total wt.% HHG mixtures prior to GDL addition.

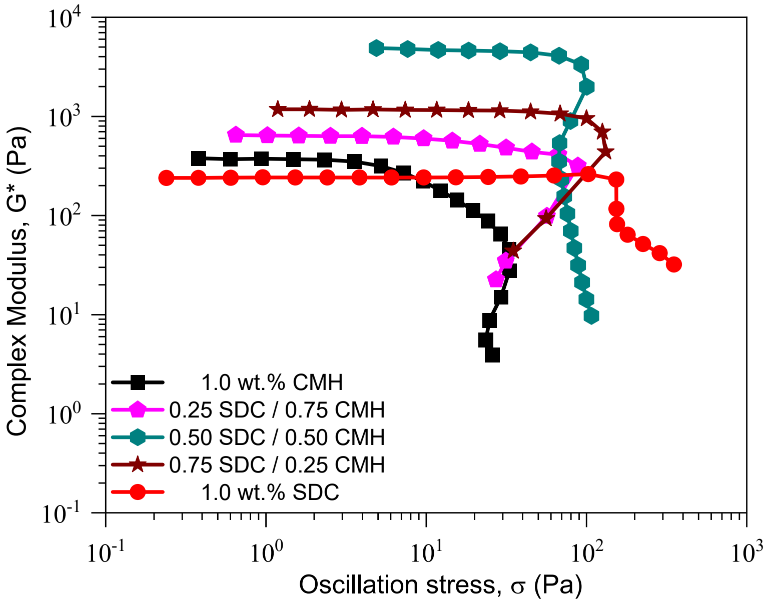

Supplementary Figure 7 | Amplitude sweeps of 1.0 wt.% total alginate HHG formulations. Cross-linked 1 wt.% HHGs of varying composition performed 2 hours after GDL addition at a frequency of 6.23 rad/s. These results show a maximum complex modulus for equilibrated HHGs is obtained at a 1/1 ratio of SDC/CMH.

Use of SDC reinforcement in double and triple "non-homocomposite" gel networks

The results suggest that SDC as additives can form the basis of strongly interconnected and efficient colloidal reinforcing networks in very broad ranges on "non-homocomposite" (multiple chemical component) hydrogels. We constructed and tested several heterocomposite gels containing SDCs networks. The results are summarized in Supplementary Figure 8.

The first series of yield stress data, shown in Supplementary Figure 8a are from gels formed by Polyvinyl Alcohol (PVOH) SDCs dispersed in Sylgard 184 PDMS liquid base. To prepare these systems, PVOH SDCs were first suspended in acetone at a concentration of 3 wt.%. This dispersion was then mixed directly with the liquid PDMS at varied concentrations and stirred magnetically at 65°C for 48 hours to remove the acetone from the mixture. The fumed silica in PDMS samples were prepared by slowly adding the Aerosil R202 particle powder to the PDMS while stirring at

65°C for 48 hours. The amplitude sweeps of SDC/PDMS composites show a 4-decade increase in the complex modulus with just 1.4 wt.% polyvinyl alcohol (PVOH) SDCs compared to the pure PDMS base. The comparison of these results with amplitude sweeps of fumed silica (Aerosil R202) in PDMS show that the SDCs far outperform the fumed silica in terms of viscoelastic modification of the continuous liquid PDMS phase even when the silica content is increased to four-fold that of the SDCs. These results highlight the extraordinary efficiency with which SDCs form physical networks within a secondary liquid medium, as fumed silica is an extremely common thickening agent for inducing thixotropy and yield stresses through a percolated, colloidal network.

We also produced "triple network" hydrogel samples, including polyacrylamide (PAAm) – SA SDC – SA CMH hydrogels. Fist, an alginate stock solution of 1.0 wt.% SA SDCs, 1.0 wt.% SA, and 0.10 wt.% $CaCO_3$ NPs was prepared. Another stock solution of 28 wt.% acrylamide, ammonium persulfate (Sigma-Aldrich) was added as the acrylamide photo initiator (0.03 wt.%), and N,N-methylenebisacrylamide (Sigma-Aldrich) was added as the crosslinker for polyacrylamide. These two stock solutions were then mixed equally by weight and stirred vigorously. To induce cross-linking of the alginate and acrylamide networks, 20 wt.% GDL in water (5/1 GDL/CaCO3 w/w) and N,N,N,N-tetramethylethylenediamine (TEMED; Sigma-Aldrich, .0025/1 TEMED/acrylamide w/w) were finally added to induce the crosslinking of both networks. The mixtures were stirred vigorously using a Vortex mixer. They were poured into 44 mm × 40 mm × 1 mm laser-cut molds, sealed using a 1.5 mm glass slide, and placed in a UV light chamber for 1 hour at 8 W power to complete cross-linking of both molecular networks.

In Supplementary Figure 8b we compare representative stress-strain curves for traditional PAAm-SA CMH double network hydrogels consisting of 14 wt.% polyacrylamide and 1 wt.% SA

CMH to a "triple network" hydrogel that maintains the total alginate concentration of at 1 wt.% with a composition of 14 wt.% PAAm, 0.5 wt.% SA CMH, 0.5 wt.% SA SDC. The inclusion of the colloidal SDC component in the "triple network" leads to increased tensile strength of the resulting gels, however, the extensibility of the resulting gels is somewhat decreased compared to the double network gels prepared without the inclusion of the colloidal SDC network.

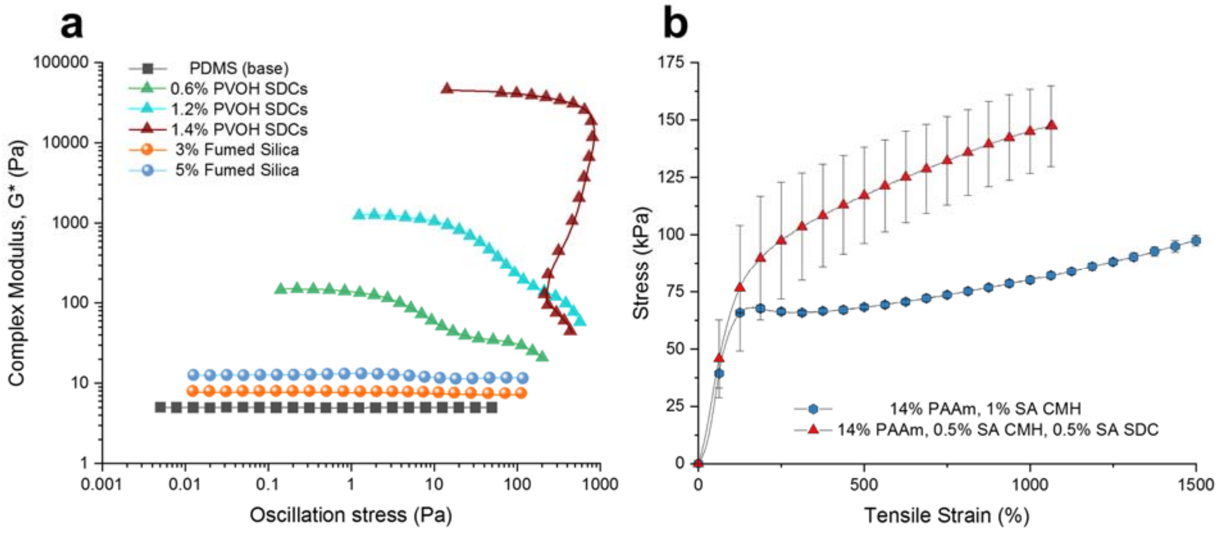

Supplementary Figure 8 | SDCs as universal composite yield stress additives. **a** Amplitude sweeps of polyvinyl alcohol (PVOH) soft dendritic colloids compared with fumed silica dispersed in PDMS base (Sylgard 184) showing yield stress emergence at low SDC concentrations (w/w). **b** Representative stress-strain curves of double network hydrogels consisting of 14% PAAm, 1% SA CMH and triple network hydrogels comprised of 14% PAAm, 0.5% SA CMH, 0.5% SA SDC (w/w) (n=3).

Experimental cells and procedures used in measurement of tensile properties of the gels

To prepare the HHGs with desired shape for tensile analysis and the syneresis measurements, laser cut acrylic sheets were used to make curing molds of desired dimensions for hydrogel curing and grippers for tensile analysis. A thin layer of cyanoacrylate adhesive (Gorilla Glue Gel) was applied at the gel-gripper interface and allowed to cure for 30 minutes prior to tensile analysis (Supplementary Fig. 9).

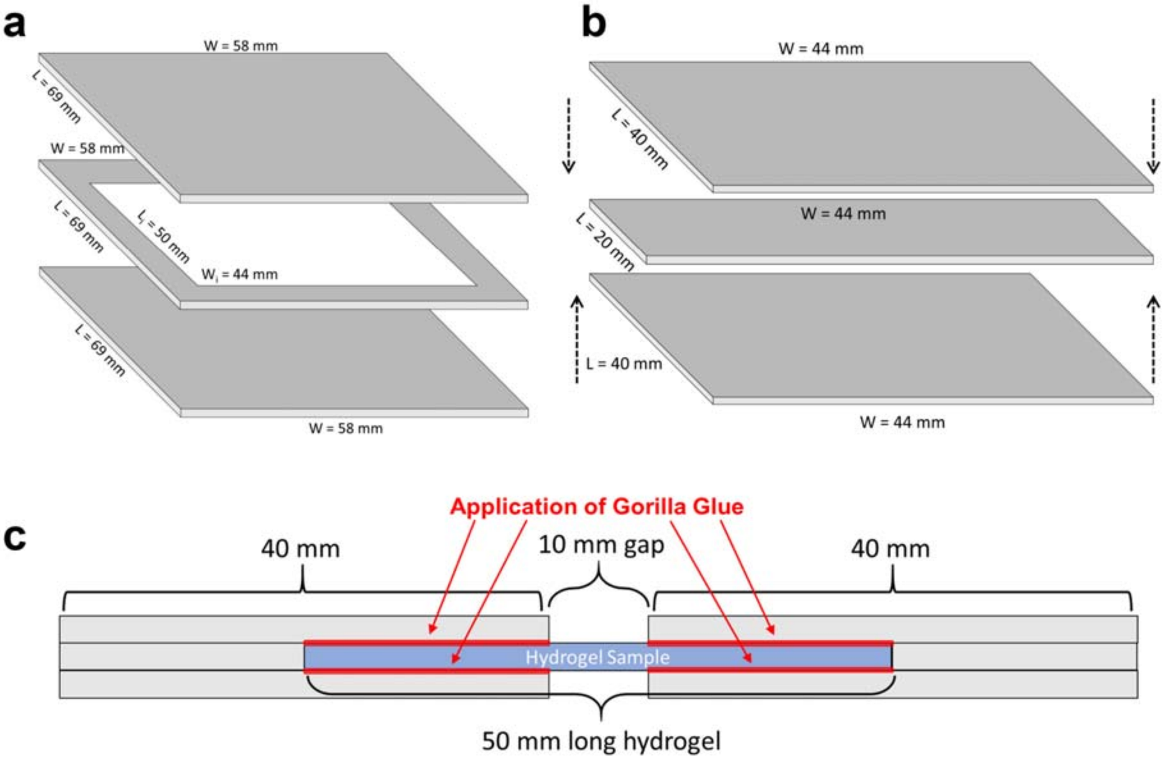

Supplementary Figure 9 | Schematic of the laser-cut acrylic **a** mold used to fabricate hydrogel sample for tensile analysis, **b** grippers used to hold the hydrogel during tensile analysis, and **c** the assembled grippers chemically bonded to the hydrogel sample with clamping zones indicated. All acrylic sheets were 1.59 mm thick and solvent-welded together using dichloromethane.
