# Supplementary material for: Printable homocomposite hydrogels with synergistically reinforced molecular-colloidal networks
Source: Nat Commun. 2021 May 14;12:2834. doi: 10.1038/s41467-021-23098-9 (PMC8121785; doi:10.1038/s41467-021-23098-9)
Supplement: Supplementary file 3 — Description of Additional Supplementary Files [file 41467_2021_23098_MOESM3_ESM.pdf]

Description of Additional Supplementary Files

File Name: Supplementary Movie 1
Description: Synergistic effect in mixed SA-SDC compositions for 3D printing. A solution of 1.5 wt.% molecular SA in H2O and a suspension of 1.5 wt.% SDC are both in fluid-like state. Once they are mixed in 1:1 ratio to yield a 1.5 wt.% homocomposite mixture, they form a gel-like extrudable system even prior to cross-linking of the molecular alginate network.

File Name: Supplementary Movie 2
Description: 3D printing via extrusion of a layered design with 1.5 wt.% SA homocomposite gel system (0.75 wt.% SDC, 0.75 wt.% CMH).
